# Supplementary material for: The Systems Biology Research Tool: evolvable open-source software
Source: BMC Syst Biol. 2008 Jun 29;2:55. doi: 10.1186/1752-0509-2-55 (PMC2446383; doi:10.1186/1752-0509-2-55)
Supplement: Additional file 1 — SBRT Archive. An archive of the current version of the Systems Biology Research Tool. [file 1752-0509-2-55-S1.zip › sbrt-1.4.0/doc/developers_guide/api/sbrt/shell/io/class-use/MapTextFile.html]

Uses of Interface sbrt.shell.io.MapTextFile


|  |  |  |
| --- | --- | --- |
| **Overview** \| **Package** \| **Class** \| **Use** \| **Tree** \| **Deprecated** \| **Index** \| **Help** | |  |
| PREV   NEXT | **FRAMES**    **NO FRAMES**     **All Classes** |  |


---


## **Uses of Interface sbrt.shell.io.MapTextFile**

| Packages that use MapTextFile | |
| --- | --- |
| **sbrt.shell.io** | Provides classes and interfaces for reading and writing files. |
| **sbrt.shell.io.fba** | Provides classes and interfaces for reading and writing files relevant to Flux Balance Analysis. |

Uses of MapTextFile in sbrt.shell.io

| Subinterfaces of MapTextFile in sbrt.shell.io | |
| --- | --- |
| `interface` | `MapTextInputFile<K,V>`             This interface is used to represent text-based input files that contain maps. |
| `interface` | `MapTextOutputFile<K,V>`             This interface is used to represent output files that maps can be written to as text. |

| Classes in sbrt.shell.io that implement MapTextFile | |
| --- | --- |
| `class` | `AbstractMapInputFile<K,V>`             This abstract class is a skeleton implemenation of text-based map input files. |
| `class` | `AbstractMapOutputFile<K,V>`             This abstract class is a skeleton implementation of `MapTextOutputFile`. |
| `class` | `ComiFile<K,V>`             This class is used to read column-oriented map input files. |
| `class` | `ComoFile<K,V>`             This class is used to write maps to text files in a column-oriented way. |
| `class` | `ProcessInputFile`             This class is used to represent process input files. |
| `class` | `RomiFile<K,V>`             This class is used to read row-oriented map input files. |
| `class` | `RomoFile<K,V>`             This class is used to write maps to text files in a row-oriented way. |

Uses of MapTextFile in sbrt.shell.io.fba

| Classes in sbrt.shell.io.fba that implement MapTextFile | |
| --- | --- |
| `class` | `FluxCapInputFile`             This class is used to read files containing flux caps. |

---


|  |  |  |
| --- | --- | --- |
| **Overview** \| **Package** \| **Class** \| **Use** \| **Tree** \| **Deprecated** \| **Index** \| **Help** | |  |
| PREV   NEXT | **FRAMES**    **NO FRAMES**     **All Classes** |  |


---
